# Draft Whole-Genome Sequence of the Type Strain *Bacillus aquimaris* TF12^T^

**DOI:** 10.1128/genomeA.00640-16

**Published:** 2016-07-14

**Authors:** Ismael L. Hernández-González, Gabriela Olmedo-Álvarez

**Affiliations:** Departamento de Ingeniería Genética, Centro de Investigacion y de Estudios Avanzados del Instituto Politécnico Nacional, Unidad Irapuato, Irapuato, Mexico

## Abstract

*Bacillus aquimaris* TF12 is a Gram-positive bacteria isolated from a tidal flat of the Yellow Sea in South Korea. We report the draft whole-genome sequence of *Bacillus aquimaris* TF12, the type strain of a set of bacteria typically associated with marine habitats and with a potentially high biotechnology value.

## GENOME ANNOUNCEMENT

In the past few decades, there has been an increasing interest in marine organisms as a possible source of bioactive compounds. *Bacillus* species have been isolated from a huge variety of environments, both terrestrial and aquatic. *Bacillus aquimaris* TF12 is the type strain of a set of bacteria typically associated with marine habitats. It is a moderately halophilic bacterium isolated from seawater of a tidal flat of the Yellow Sea in South Korea (1). Strains of *B. aquimaris* have been studied as a possible source of bioactive compounds, such as starch-degrading enzymes (2), proteases (3, 4), and organic solvent stable alkaline cellulases (5).

The type strain of *B. aquimaris* TF12 was obtained from DSMZ (Deutsche Sammlung von Mikroorganismen und Zellkulturen GmbH). Its genome was sequenced using a hybrid method. The raw reads were obtained using 454 GS-FLX and Illumina mate-pair sequencing. The 454 reads were processed using the Newbler software, while Illumina reads were trimmed and filtered by quality and length employing the FastX-toolkit (http://hannonlab.cshl.edu/fastx_toolkit/index.html). The *de novo* assembly was performed using MIRA3 (6) and Velvet (7). The final assembly resulted in 30 contigs (>1000 bp) that comprise 4,035,445 bases with a G+C content of 37.3%. Genome annotation was done employing RAST (Rapid Annotation using Subsystem Technology) (8). It contains 4,184 open reading frames (ORF) and 23 rRNA and 83 tRNA genes. Almost 21.2% of the ORFs were hypothetical proteins (1,013 ORFs). The phylogenetic reconstruction using the 16S rRNA gene showed that the closest strain to *B. aquimaris* TF12 was *Bacillus coahuilensis* m4-4 (9), with which it shares 25% of orthologs genes.

The annotation of the genome through RAST showed that *B. aquimaris* had numerous genes for amino acid and carbohydrate metabolism, followed by cofactors, vitamins, prosthetic groups, and pigments subsystems. Puspasari et al. (2012) (2) reported an α-amylase isolated from *B. aquimaris* MKSC 2.6 which together with the sequences of other *Bacillus* species defined a new sub-family of α-amylases (GH13) (2). To verify if *B. aquimaris* TF12 carried a homologous sequence to this α-amylase, we did a search in the genome of *Bacillus aquimaris* TF12 using the amino acids sequence of the BaqA gene (accession no. JN797599). Although the best hit showed 84% identity with the BaqA sequence, it exhibited the motif of two consecutive tryptophan residues that defines the proposed subfamily.

The search for possible proteases using the MEROPS BLAST server (MEROPS v9.13) (10) identified 172 proteins classified in 9 families. The families of proteolytic protein most represented are those comprising metallo peptidases (72), followed by the family of serine peptidases (61), cysteine peptidases (24), aspartic peptidases (4), peptidase inhibitors (4), peptidases of unknown catalytic type (3), asparagine peptidases (2), a threonine peptidase, and a mixed peptidase.

Furthermore, relevant to the importance of this species as a producer of compounds of biotechnological value, *B. aquimaris* and its relatives represent one new group inside the *Bacillus* genus*.* The knowledge obtained from the study of the genome of this organism would shed light on the evolution of the *Bacillus* genus*.*

### Nucleotide sequence accession numbers.

This whole-genome shotgun project has been deposited in DDBJ/EMBL/GenBank under the accession no. LQXM00000000. The version described in this paper is the first version, LQXM01000000.
